# 

**Published:** 1883-12

**Authors:** 


					﻿Whole Number,
/CCCCLXVII.
December, 1883.
Number 6,
Vol. XLVIL
THE
CHICAGO
MEDICAL
Journal & Examiner.
CHICAGO: "
PUBLISHED BY THE CHICAGO MEDICAL PRESS ASSOCIATION
188 Clark Street.
^"Read the Notice of this Journal among Advertisements
				

